# Effects of Diethylstilbestrol on Uterus Structure and Immunological Function in Mice During Early Pregnancy

**DOI:** 10.3390/toxics13080672

**Published:** 2025-08-09

**Authors:** Jian Li, Ruiping Xu, Guan Wang, Yanhua Su, Yaoxing Chen, Jing Cao

**Affiliations:** 1 Anhui Province Key Laboratory of Embryo Development and Reproductive Regulation, College of Biology and Food Engineering, Fuyang Normal University, Yingzhou, Fuyang 236037, China; lijian800702@126.com; 2 Laboratory of Anatomy of Domestic Animals, State Key Laboratory of Veterinary Public Health and Safety, College of Veterinary Medicine, China Agricultural University, Haidian, Beijing 100193, China; xrp15237375652@163.com (R.X.); 19503887765@163.com (G.W.); yxchen@cau.edu.cn (Y.C.); 3 College of Veterinary Medicine, Yunnan Agricultural University, Panlong, Kunming 650201, China; 2013003@ynau.edu.cn

**Keywords:** diethylstilbestrol, pregnant mice, uterine glands, oxidative stress, immunity

## Abstract

Due to the growing environmental burden of endocrine-disrupting chemicals (EDCs), there is an increasing concern regarding the reproductive hazards posed by synthetic estrogens, particularly diethylstilbestrol (DES). However, the precise mechanisms by which DES disrupts uterine endocrine function and immune homeostasis leading to pregnancy failure remain unclear. Given that wild rodents serve as key reservoirs for zoonotic diseases such as plague, reproductive interventions targeting their pregnancy processes hold significant ecological implications for disease control. In this study, female mice in estrus were randomly divided into four experimental groups, receiving DES at doses of 0 (control), 0.02 (low), 0.2 (medium), and 2 mg/kg (high), respectively. For five consecutive days, mice were injected subcutaneously on a daily basis, with the goal of examining DES-related alterations in hormone secretion and local immune responses within the uterus and spleen. It was found that high-dose DES treatment significantly increased maternal body weight and spleen weight during early pregnancy (*p* < 0.05). Meanwhile, reproductive function declined progressively with increasing doses, as indicated by decreased ovarian and uterine weights, fewer embryos, and extended estrous cycle duration (*p* < 0.05). Hematoxylin and eosin staining revealed that high-dose DES markedly reduced uterine gland density at day P5, accompanied by epithelial vacuolar degeneration and nuclear pyknosis. The proportion of uterine glands relative to total uterine area also decreased significantly with increasing DES doses. Moreover, DES inhibited lymphocyte proliferation in both the uterus and spleen in a dose-dependent fashion, with ConA- and LPS-induced proliferation rates decreasing by 0.78–30.70% and 1.91–18.20%, respectively (*p* < 0.05). The proinflammatory cytokine IL-2 was significantly elevated by DES, whereas the anti-inflammatory cytokine IL-4 showed a notable decrease (*p* < 0.05). DES administration notably decreased uterine expression of proliferating cell nuclear antigen. In contrast, the numbers of B-cell lymphoma 2- and Bcl-2-associated X protein-positive cells rose, along with upregulated levels of inducible nitric oxide synthase. Furthermore, DES impaired antioxidant defenses in both the uterus and spleen, evidenced by the decreased activities of superoxide dismutase and glutathione peroxidase, reduced total antioxidant capacity, and elevated malondialdehyde levels. This study elucidates the multifaceted mechanisms by which DES impairs the early gestational reproductive environment, filling a critical knowledge gap regarding its interference with the uterus–immune axis, and expands the current understanding of the ecotoxicological impacts of endocrine-disrupting chemicals.

## 1. Introduction

Rodents act as natural reservoirs for a broad spectrum of zoonotic diseases, particularly plague, and are instrumental in maintaining and disseminating pathogens within endemic foci [1]. Effective rodent population control is widely recognized as a crucial public health strategy to mitigate disease transmission [2]. However, conventional approaches—such as chemical rodenticides and physical trapping—are often costly, prone to resistance development, and pose ecological risks to non-target species, thereby limiting their sustainability and specificity for long-term management [3]. In recent years, ecological control has emerged as a more targeted and sustainable alternative. This strategy aims at regulating populations by interfering with reproductive processes, while minimizing ecological disturbance [4,5]. Researchers have also investigated the use of synthetic hormones and environmental endocrine-disrupting chemicals (EDCs) to suppress animal fertility. Hormonal agents, including norgestrel and levonorgestrel, have been widely used to manage reproduction in captive animals, livestock, and wildlife [6,7].

Among available fertility control strategies, targeting the pregnancy stage is particularly appealing due to its narrow and well-defined window of intervention [8,9]. Pregnancy represents a highly sensitive phase in the mammalian reproductive cycle and is especially vulnerable to exogenous perturbations. Successful gestation depends on the finely tuned regulation of uterine hormone signaling, immune tolerance, and redox homeostasis [10,11,12]. Environmental EDCs can impair these microenvironments, ultimately leading to implantation failure or pregnancy loss [13]. Diethylstilbestrol (DES), a synthetic estrogen once prescribed to prevent miscarriage, is a well-known endocrine disruptor capable of inducing the abnormal proliferation of uterine epithelial cells, triggering Wnt/β-catenin and PI3K/AKT signaling cascades, and intensifying inflammatory responses, ultimately impairing endometrial receptivity [14,15]. However, the temporal dynamics and mechanistic basis of DES action during early pregnancy remain inadequately understood.

Given that wild rodents are key hosts of plague and other infectious diseases, reproductive interventions targeting the pregnancy stage offer a promising ecological approach to rodent population management and disease control [16]. In this study, mice were utilized to comprehensively assess how DES exposure influences major reproductive indicators—including serum hormone concentrations, uterine histological structure, immune activity at the tissue level, cellular growth and death, and oxidative stress—at five pivotal stages of early pregnancy (days 1, 3, 5, 7, and 9; designated P1, P3, P5, P7, and P9). This comprehensive investigation aims at elucidating the mechanisms by which DES disrupts uterine function and identifying time-dependent vulnerabilities to endocrine perturbation during early gestation. These findings are expected to provide both mechanistic insight and temporal targets for precision reproductive regulation in rodents, thereby supporting ecological control strategies for plague and other rodent-borne diseases.

## 2. Materials and Methods

### 2.1. Animal Husbandry and Tissue Collection

A total of 120 adult CD-1 mice (8 weeks old) were used, consisting of 100 females (25–33 g) and 20 breeding males. All animals were supplied by Beijing Vital River Laboratory Animal Technology Co., Ltd. They were maintained in individually ventilated cages (IVCs; dimensions 30 × 20 × 15 cm, ≈600 cm^2^ floor area, up to five mice per cage) following the National Laboratory Animal Standard (GB 14925-2010). Conditions were set to maintain temperatures within 23–25 °C, relative humidity between 65% and 70%, and a ventilation frequency of 15 to 20 air exchanges each hour. Lighting was maintained for 14 h each day, followed by 10 h of darkness. The lights were turned on at 06:00 and switched off at 20:00. Corncob bedding was changed twice weekly, and nesting materials were provided as enrichment. During the 7-day acclimation phase, mice were given a regular chow diet, with sterilized water provided *ad libitum*. Subsequently, the females were randomly assigned into four groups (*n* = 25) using a computer-generated algorithm. The distribution ensured similar initial body weights and estrous cycle stages among groups: one control group treated with olive oil (0 mg/kg) and three groups receiving diethylstilbestrol at low (0.02 mg/kg), medium (0.2 mg/kg), or high (2 mg/kg) doses, respectively. DES purchased from Sigma-Aldrich, (Darmstadt, Germany) was freshly made into a solution using olive oil (Med-ChemExpress, Shanghai, China) at specified concentrations prior to injection. Each mouse received a subcutaneous dose of 0.1 mL, tailored according to its body weight measured on the day of administration. Injections were administered at the same time each day for five days during the estrus phase, which was determined by daily vaginal smear cytology under microscopic examination. Two days after the final injection, estrus was re-confirmed using the same method, and females in estrus were paired with males in clean cages overnight. The presence of a copulatory vaginal plug was observed under adequate lighting at 7:00 a.m. the next day served as a confirmation of successful mating, with that day designated as pregnancy day 1 (P1). To reduce variability caused by circadian rhythms, body weights were recorded with an electronic balance on gestation days P1, P3, P5, P7, and P9 consistently at a fixed time each day. On P9, mice were intraperitoneally administered sodium pentobarbital (50 mg/kg) for anesthesia, followed by blood collection through retro-orbital bleeding. The animals were then sacrificed via cervical dislocation. An incision was made along the midline to access the abdominal cavity, after which periuterine and periovarian fat was carefully removed. The uterus and ovaries were subsequently isolated, rinsed, and weighed. Embryo counts in gravid uteri were performed under a dissecting microscope. Tissue samples were processed in two ways: one portion was fixed in 4% paraformaldehyde (pH 7.4) from Beyotime (Shanghai, China) phosphate-buffer solution (pH 7.4) for histological analysis, while the other was snap-frozen at −80 °C for further analyses.

### 2.2. Serum Hormone and Cytokine Measurement

Following blood collection, the samples were kept at 4 °C for 2 h and then centrifuged at 3500 rpm for 10 min to isolate the serum. Estradiol, progesterone, interleukin-2 (IL-2), and interleukin-4 (IL-4) levels were measured using ELISA kits (Elabscience, Wuhan, China), following the provided protocols [17]. Using a BIO-RAD microplate reader (Hercules, CA, USA), absorbance at 450 nm was recorded. Concentrations were then derived from standard curves and expressed in pg/mL.

### 2.3. Hematoxylin and Eosin Staining

Uterine samples were initially immersed in 4% paraformaldehyde (pH 7.4, Beyotime, Shanghai, China) for fixation. Afterwards, they underwent stepwise dehydration in graded ethanol (Beyotime, Shanghai, China), followed by clearing in xylene and embedding in paraffin wax (Sinopharm, Beijing, China). Thin sections measuring 5 μm in thickness were prepared, followed by deparaffinization and rehydration before further analysis. Sections (5 μm thick) were prepared from paraffin-embedded tissue blocks and underwent deparaffinization and rehydration before further procedures. Sections were stained for 20 min using Ehrlich’s hematoxylin (Beyotime, Shanghai, China), followed by acid alcohol differentiation, bluing with tap water, and 1% eosin counterstaining. Uterine morphology was evaluated under a light microscope (Olympus BX51, Tokyo, Japan). Image analysis software (Image-Pro Plus 6.0, Media Cybernetics, Rockville, MD, USA) was used to quantify the glandular area, which was then presented as a percentage of the entire uterine region.

### 2.4. Lymphocyte Proliferation Assay

Lymphocyte proliferation in uterine and splenic tissues was assessed via the MTT assay following stimulation with concanavalin A (Con A, Sigma-Aldrich, Darmstadt, Germany) or lipopolysaccharide (LPS, Sigma-Aldrich, Darmstadt, Germany) [18]. Single-cell suspensions were obtained by passing tissues through a nylon mesh under sterile conditions. Using Trypan blue exclusion (Sigma-Aldrich, Darmstadt, Germany), cell viability was evaluated, then the suspension was diluted to 1 × 10^5^ cells/mL. Next, after plating cells in 96-well plates, treatments with concanavalin A or lipopolysaccharide were applied at final doses of 5, 10, or 20 µg/mL. Each condition was performed in triplicate with appropriate controls. Plates were incubated for 48 h at 37 °C in a humidified 5% CO_2_ incubator (Thermo Fisher Scientific, Waltham, MA, USA). Subsequently, the mixture was treated with 10% SDS and left to react for an additional 2 h. Proliferative activity was determined based on absorbance readings at 570 nm obtained with a microplate reader.

### 2.5. Immunohistochemistry

Paraffin-embedded uterine tissue sections (5 μm) underwent deparaffinization and rehydration, followed by three rinses in 0.01 mol/L PBS (each lasting 5 min). Antigen unmasking was subsequently conducted using microwave treatment. To inhibit endogenous peroxidase activity, tissue sections were incubated with 3% hydrogen peroxide for 30 min at room temperature, then blocked with 10% fetal calf serum for another 30 min to prevent non-specific binding. The tissues were exposed to the following respective primary antibodies and kept at 4 °C overnight: anti-proliferating cell nuclear antigen (PCNA, mouse monoclonal, 1:5000, Sigma-Aldrich, Darmstadt, Germany), anti-B-cell lymphoma 2 (Bcl-2, rabbit polyclonal, 1:50, Boster Biological Technology Co., Ltd., Wuhan, China), anti- Bcl-2-associated X protein (Bax, rabbit polyclonal, 1:100, Boster Biological Technology Co., Ltd., Wuhan, China)**,** and anti-inducible Nitric Oxide Synthase (iNOS, rabbit polyclonal, 1:200; all from Zhongshan Golden Bridge, Beijing, China). Biotinylated secondary antibodies—specific to PCNA (horse anti-mouse IgG, Zhongshan Golden Bridge, Beijing, China) and other targets (goat anti-rabbit IgG, Zhongshan Golden Bridge, Beijing, China)—were applied, followed by a 1.5 h incubation with HRP-conjugated streptavidin (1:300; Zhongshan Golden Bridge, Beijing, China). After washing with PBS three times, 0.05% DAB was added for color development for 2–5 min. After counterstaining with hematoxylin, the sections underwent dehydration and clearing before being mounted. Negative controls were processed in parallel, substituting PBS for the primary antibody, with no alterations to subsequent procedures. For each group, five sections per animal were randomly selected, and five fields per section were randomly chosen for microscopic imaging. Positively stained cell counts per unit area were analyzed with Image-Pro Plus 6.0 software (Media Cybernetics, Rockville, MD, USA).

### 2.6. Antioxidant Index Assays

The activities of total superoxide dismutase (T-SOD), glutathione peroxidase (GSH-Px), total antioxidant capacity (T-AOC), and malondialdehyde (MDA) in uterine and splenic tissues were measured with assay kits purchased from Nanjing Jiancheng Bio-engineering Institute (Nanjing, China), following the protocols provided by the manufacturer. Tissue homogenates (prepared in 0.9% pre-chilled saline at a 1:9 ratio) were centrifuged at 3000 rpm for 10 min at 4 °C. The clear supernatants obtained after centrifugation were harvested for further use. Reagents were prepared and incubated as instructed by the kit protocols, and antioxidant indices were quantified by recording absorbance at designated wavelengths with a spectrophotometer.

### 2.7. Statistical Analysis

Data statistical analyses were carried out with SPSS version 20 (SPSS Inc., Chicago, IL, USA). A one-way ANOVA was performed to evaluate the effect of DES dose (0, 0.02, 0.2, and 2 mg/kg) as the main factor. Data were first tested for normality with the Shapiro–Wilk test, followed by assessments of independence and variance homogeneity using Levene’s test before further analysis. All values are shown as mean ± SD, and significance was assigned to comparisons yielding *p* < 0.05.

## 3. Results

### 3.1. DES-Induced Changes in Maternal Body Weight, Organ Development, and Embryo Number

**Figure 1:**
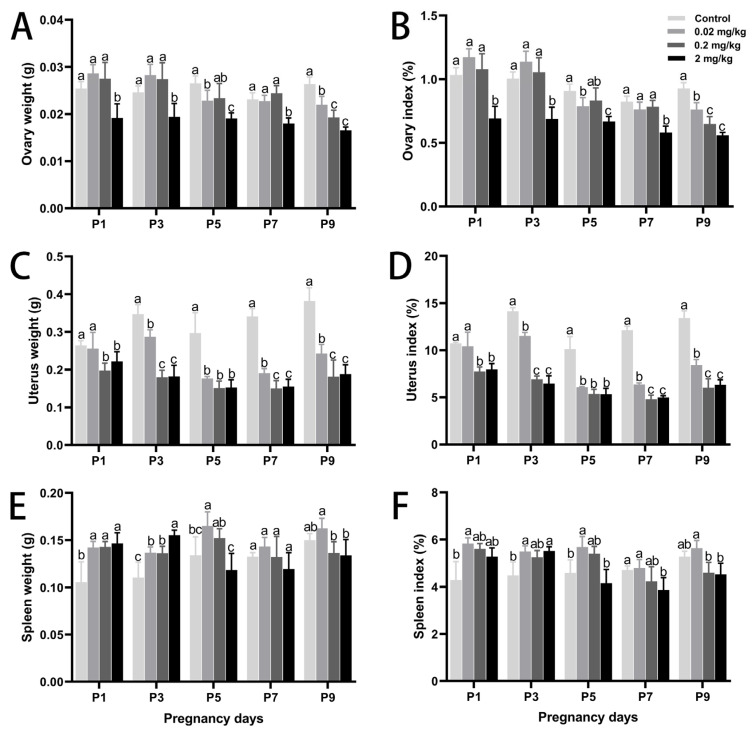
Effects of DES treatment on organ development and organ coefficients in pregnant mice. (**A**,**B**) Ovary weight and ovary coefficient. (**C**,**D**) Uterus weight and uterus coefficient. (**E**,**F**) Spleen weight and spleen coefficient. Groups labeled with different superscript letters exhibit significant differences (*p* < 0.05); the same letter indicates no significant difference (*p* > 0.05). Data are reported as mean ± SD.

DES exposure significantly impaired reproductive physiology in mice during early pregnancy, as evidenced by abnormal maternal weight gain, reduced reproductive organ weights, and decreased embryo numbers. These effects exhibited both dose- and time-dependent trends (Table 1 and Figure 1). Relative to controls, maternal body weights in the high-dose DES (2 mg/kg) group increased significantly by 11.80% on P1 and 9.40% on P3 (*p* < 0.05). Comparable patterns appeared at other time points but did not reach statistical significance. Notably, DES significantly suppressed uterine and ovarian development. Organ weights and relative indices progressively declined with increasing DES doses and advancing gestation. On P9, uterine and ovarian weights in the high-dose group were reduced by 35.20% and 28.60%, respectively, relative to controls (*p* < 0.05). Moreover, the estrous cycle was lengthened by 0.6, 1.5, and 2.7 days in the groups receiving low, medium, and high doses of DES, respectively. Embryo counts were significantly lower in the high-dose group, averaging approximately 9.2 fewer embryos per mouse compared to controls (*p* < 0.05). Interestingly, spleen development displayed a stage-dependent response to DES. On P1 and P3, spleen weight and index increased in a dose-dependent manner. Starting on P5, the high-dose group showed notable decreases, reaching a maximum on P9 where spleen weight and index dropped by 8.96% and 14.31%, respectively, without notable differences (*p* > 0.05).

### 3.2. DES-Induced Changes in Uterine Histology and Serum Hormone Levels

**Figure 2:**
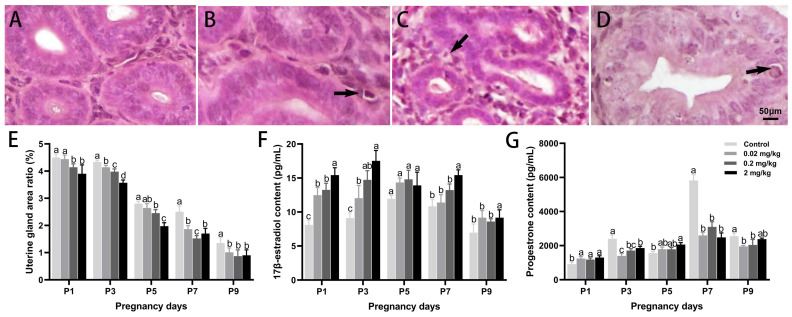
Effects of DES treatment on the histological structure of uterine glandular epithelium and serum hormone levels in pregnant mice. (**A**–**D**) Representative images of the control, 0.02 mg/kg, 0.2 mg/kg, and 2 mg/kg groups, respectively. Scale bar = 50 µm. Arrows indicate vacuolated cells. (**E**) Percentage of uterine gland area relative to the total uterine area. (**F**) Serum levels of 17β-estradiol. (**G**) Serum levels of progesterone. Groups labeled with different superscript letters exhibit significant differences (*p* < 0.05); the same letter indicates no significant difference (*p* > 0.05). Data are reported as mean ± SD.

Hematoxylin and eosin staining revealed marked DES-induced histological alterations in uterine glands in a dose-dependent manner (Figure 2). On P5, medium- and high-dose groups displayed disrupted glandular architecture, epithelial vacuolation, and nuclear pyknosis. Uterine gland density significantly declined with DES exposure. Compared to controls, the high-dose group exhibited reductions of 13.37%, 17.84%, 29.74%, and 33.75% on P1, P3, P5, and P9, respectively (*p* < 0.05), while an increase of 46.59% was observed on P7 (*p* < 0.05). Serum hormone profiling showed persistent elevations in 17β-estradiol levels in the high-dose group across all time points, with the most substantial rise on P3 (92.57% above control; *p* < 0.05). Conversely, progesterone levels did not show consistent dose- or time-dependent changes but were significantly reduced on P3, P7, and P9 in all DES-treated groups (*p* < 0.05).

### 3.3. DES-Induced Changes in Local Immune Responses in the Uterus and Spleen

**Figure 3:**
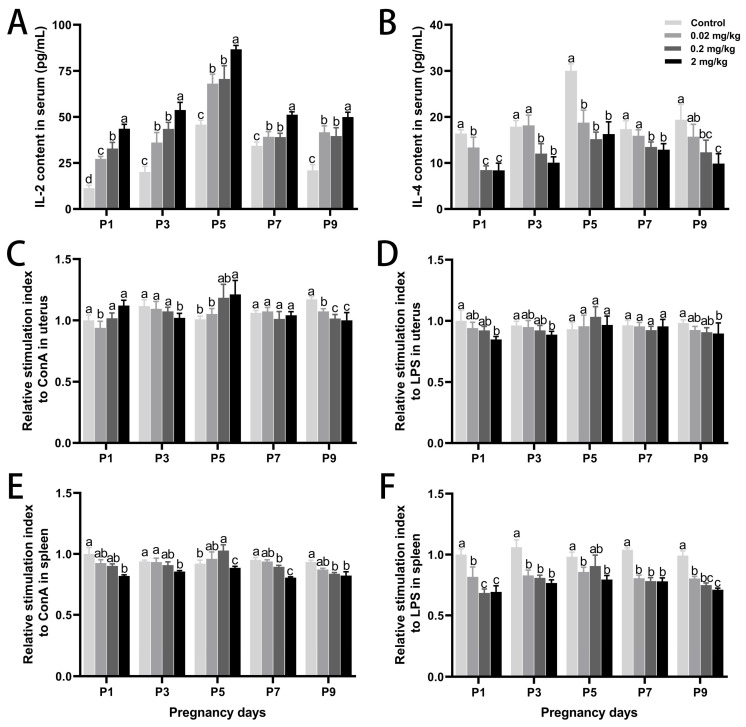
Effects of DES treatment on peripheral blood cytokines and lymphocyte proliferation in pregnant mice. (**A**,**B**) IL-2 and IL-4 concentrations in peripheral blood. (**C**,**D**) Proliferative responses of uterine lymphocytes to Con A and LPS. (**E**,**F**) Proliferative responses of splenic lymphocytes to Con A and LPS. Groups labeled with different superscript letters exhibit significant differences (*p* < 0.05); the same letter indicates no significant difference (*p* > 0.05). Data are reported as mean ± SD.

To investigate DES-induced immunomodulation during early pregnancy, serum levels of Th1/Th2 cytokines were assessed (Figure 3). DES disrupted the Th1/Th2 balance, as IL-2 levels increased dose-dependently, with the highest elevation on P5 (89.21% increase vs. control; *p* < 0.05) in the high-dose group. In contrast, IL-4 levels declined across all DES groups, with a maximum reduction of 45.85% on P5 (*p* < 0.05). Additionally, DES significantly inhibited lymphocyte proliferation in both uterine and splenic tissues. This immunosuppressive effect was most prominent in the high-dose group. After Con A or LPS stimulation, all DES-treated groups exhibited reduced lymphocyte proliferation (*p* < 0.05). On P9, the stimulation indices of uterine and splenic lymphocytes in the high-dose group declined by 27.97% and 28.30% under LPS stimulation and by 14.60% and 12.00% under Con A stimulation, respectively (*p* < 0.05).

### 3.4. DES-Induced Changes in Cell Proliferation, Apoptosis, and Oxidative Stress in the Uterus

**Figure 4:**
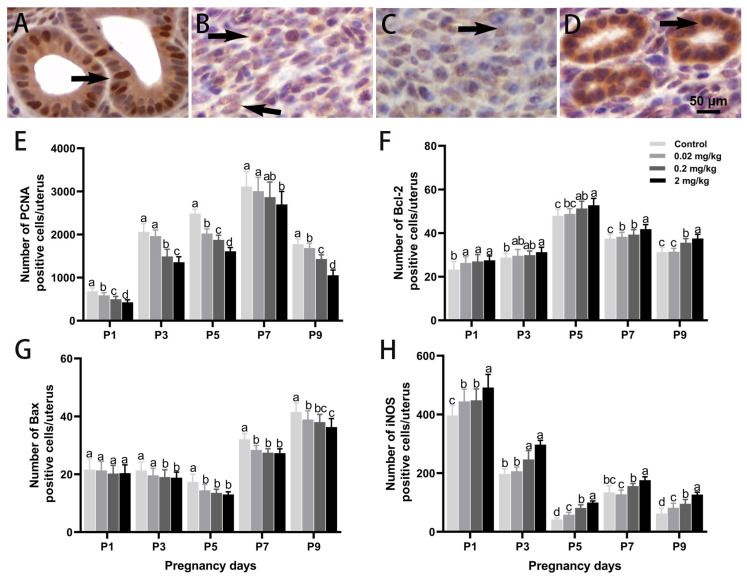
Effects of DES treatment on the expression of PCNA, Bcl-2, Bax, and iNOS in the uterine glandular epithelium of pregnant mice. (**A**,**E**) PCNA expression. (**B**,**F**) Bcl-2 expression. (**C**,**G**) Bax expression. (**D**,**H**) iNOS expression. Arrows point to cells showing positive immunostaining. Scale bar = 50 µm. Groups labeled with different superscript letters exhibit significant differences (*p* < 0.05); the same letter indicates no significant difference (*p* > 0.05). Data are reported as mean ± SD.

Immunohistochemistry demonstrated spatially distinct, dose-responsive changes in the uterine expression of PCNA, Bcl-2, Bax, and iNOS during early pregnancy (Figure 4). PCNA was predominantly localized in the nucleus. A significant, dose-dependent suppression of PCNA expression was observed following DES treatment, with the high-dose group on P9 showing a 40.73% reduction compared to controls (*p* < 0.05). Bcl-2 levels exhibited a non-linear pattern, increasing and then declining with gestational progression, and peaking on P5. Compared to the control group, Bcl-2 levels increased by 1.72% (*p* > 0.05), 6.89% (*p* < 0.05), and 9.98% (*p* < 0.05) in the low-, medium-, and high-dose DES groups, respectively. In contrast, Bax expression showed a general downward trend, especially at medium and high DES doses. At five time points, Bax levels were reduced by 5.65% (*p* > 0.05), 11.73% (*p* < 0.05), 25.08% (*p* < 0.05), 14.98% (*p* < 0.05), and 12.60% (*p* < 0.05) relative to the control. iNOS was primarily localized in the cytoplasm. While its expression naturally declined throughout over the course of gestation, DES treatment induced a sustained upregulation at multiple time points. In the high-dose group, iNOS-positive cell counts increased by 23.99%, 50.28%, 137.52%, 30.36%, and 102.70% (*p* < 0.05), respectively, compared with the control.

### 3.5. DES-Induced Changes in Antioxidant Capacity of Uterine and Splenic Tissues

**Figure 5:**
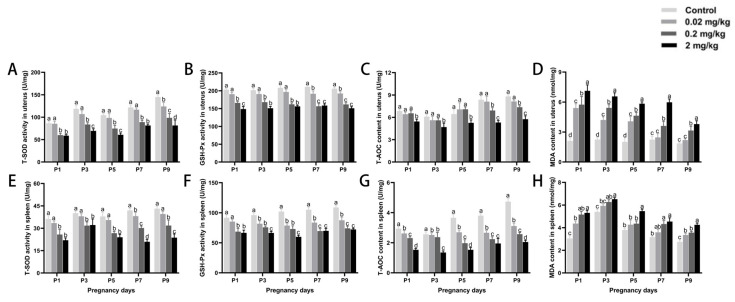
Effects of DES treatment on uterine and splenic antioxidant status in pregnant mice. (**A**–**D**) Activities of T-SOD, GSH-Px, T-AOC, and MDA in the uterus. (**E**–**H**) Activities of T-SOD, GSH-Px, T-AOC, and MDA in the spleen. Groups labeled with different superscript letters exhibit significant differences (*p* < 0.05); the same letter indicates no significant difference (*p* > 0.05). Data are reported as mean ± SD.

Exposure to DES caused a concentration-dependent decrease in antioxidant levels within the uterus and spleen (Figure 5). T-AOC, GSH-Px, and T-SOD activities in these organs declined significantly with increasing DES doses at every gestational time point assessed. Compared with controls, T-SOD activity on P9 was reduced by 43.80% in the uterus and 45.05% in the spleen of mice treated with high-dose DES (*p* < 0.05). Similarly, GSH-Px activity declined by 26.77% and 33.60% and T-AOC activity dropped by 39.81% and 56.62% (*p* < 0.05) in the uterus and spleen, respectively. In contrast, in comparison with the control group, MDA levels increased significantly with both advancing gestational age and rising DES exposure. The highest MDA concentrations were observed in the uterus on P1 and in the spleen on P2 in the high-dose group, representing increases of 237.10% and 74.00% (*p* < 0.05), respectively.

## 4. Discussion

As important natural reservoirs of plague, rodents possess a high reproductive capacity, which is a key factor supporting the persistence and transmission of the pathogen in the wild [1]. However, traditional chemical rodenticides are increasingly constrained by issues such as resistance development, non-target toxicity, and ecological disruption [19]. Therefore, alternative strategies with reduced ecological impact and sustained regulatory efficacy are urgently required. In this context, early-pregnant mice were used in this study as a model to systematically evaluate the effects of DES, a typical environmental estrogen, on immune responses and antioxidant capacity in the uterus and spleen, with the aim of elucidating the mechanisms by which DES disrupts reproduction and pregnancy maintenance.

Early pregnancy is a critical period during which the maternal organism undergoes substantial physiological adaptations to support embryo implantation and development. These changes include maternal weight gain and the remodeling of reproductive organs such as the uterus and ovaries [20]. Weight gain reflects energy storage and metabolic shifts required to sustain embryonic growth [21]. At the same time, the uterus undergoes extensive tissue remodeling for implantation, while the ovaries maintain progesterone production via the corpus luteum—both essential for successful pregnancy establishment and maintenance [22,23]. Previous studies have demonstrated that hormone-mediated changes in uterine and ovarian weight significantly influence uterine receptivity and embryonic survival [24,25]. This study demonstrated that DES exposure caused dose-dependent decreases in maternal weight gain, reproductive organ weights, and embryo numbers. These findings suggest that high-dose DES impairs maternal metabolic adaptation, disrupts uterine preparedness, and interferes with ovarian endocrine function, ultimately compromising embryo implantation and early development. These outcomes are consistent with prior reports of DES-induced ovarian atrophy and embryonic loss in rodent models [15].

Ovarian dysfunction is often accompanied by hormonal imbalances [26]. Our data show that DES exposure elevated uterine estradiol and decreased progesterone levels, particularly in the high-dose group, thereby disrupting the critical estradiol/progesterone balance required for successful implantation. Hormonal imbalance may lead to premature endometrial transformation, impaired maternal–fetal signaling, and subsequent implantation failure or early embryonic loss [27]. Estrogens and progesterone also regulate endometrial proliferation and differentiation via pathways associated with the cell cycle and apoptosis [28,29,30]. In this study, DES treatment significantly decreased PCNA expression, indicating impaired cell proliferation. The concurrent upregulation of Caspase-3 and Bax, along with the downregulation of Bcl-2, indicated enhanced apoptotic activity. This imbalance likely compromised endometrial integrity and receptivity, negatively impacting embryo implantation and pregnancy maintenance. The hormonal control of uterine activity involves not only local feedback but also systemic regulation through the hypothalamic-–pituitary-–gonadal (HPG) axis [31,32]. This axis governs gonadotropin release, ovarian steroidogenesis, and ovulatory cycles, thereby coordinating reproductive physiology [33]. In this study, DES exposure prolonged the estrous cycle in a dose-dependent manner, implying potential interference with the HPG axis function and its downstream reproductive processes, including ovulation and implantation.

Pregnancy is governed by both hormonal and immunological mechanisms [34]. Successful implantation and fetal development require the maternal immune system to tolerate paternal antigens while remaining responsive to pathogens [35]. Spleen, as the largest peripheral immune organ, dynamically modulates its immune profile during pregnancy. Cytokine secretion and lymphocyte activity are key indicators of immune status [36,37]. Cytokines such as IL-2 and IL-4 play essential roles in immune regulation. IL-2 promotes T cell proliferation and is critical for maternal–fetal immune tolerance [38,39], while IL-4 supports anti-inflammatory responses and immune balance [40]. Our results show that DES significantly suppressed lymphocyte proliferation and altered cytokine expression patterns. Specifically, IL-2 levels were downregulated and IL-4 levels were upregulated in a dose-dependent manner, indicating a shift toward humoral immunity and heightened inflammatory potential. Such dysregulation could weaken maternal immunological support for the embryo and increase susceptibility to immune-mediated embryonic loss [41].

The control of local oxidative stress and inflammatory responses within the uterus is equally vital during pregnancy for maintaining uterine homeostasis and supporting proper embryo implantation and growth [42]. Therefore, changes in uterine antioxidant capacity and iNOS-positive cells were further analyzed to determine the influence of DES on intrauterine conditions and embryo development. During pregnancy, reactive oxygen species (ROS) levels increase, which may lead to oxidative stress, causing damage to uterine tissues and destabilizing the intrauterine environment, ultimately hindering embryo implantation and development [42,43]. Endogenous enzymes, including SOD, CAT, and GSH-Px, contribute to redox balance by scavenging excess ROS [44]. In parallel, iNOS contributes to inflammatory responses through nitric oxide production, which facilitates blood flow and supports immune defense. However, the overexpression of iNOS may intensify oxidative stress and inflammation, ultimately causing uterine damage and interfering with normal embryonic development and pregnancy maintenance [45]. Our results showed that, in early pregnancy, DES exposure remarkably reduced antioxidant markers (GSH-Px, T-SOD, and T-AOC) in both the uterus and spleen, with high doses exerting the strongest effects. Meanwhile, MDA levels and the quantity of iNOS-positive cells in the uterus showed a dose-dependent increase. The results indicate that DES intensifies both oxidative injury and immune-related inflammation in uterine and splenic tissues. Earlier research has shown a strong link between increased oxidative stress during pregnancy and both impaired embryonic development and a heightened miscarriage risk [46,47]. Therefore, from a plague prevention standpoint, this non-lethal ‘covert damage’ mechanism—driven by oxidative stress and immune suppression—supports the design of innovative fertility regulation approaches. However, since our study used laboratory CD-1 mice, translating these findings to wild rodent populations may be limited by differences in genetics, environment, and ecological factors. Further research using wild rodents in natural environments is required to confirm and improve the ecological applicability of our findings.

## 5. Conclusions

DES disrupts early pregnancy in mice via multiple mechanisms, including altered maternal weight gain, impaired reproductive organ development, local immune suppression, increased apoptosis, and oxidative stress. These effects are dose-dependent and collectively reduce reproductive success. The multi-target action of DES highlights its potential as a biological tool for rodent fertility control and zoonotic disease prevention. Further research is necessary to assess its safety profile and ecological implications before considering its application in public health and environmental management.

## Figures and Tables

**Table 1:** Influence of different DES exposure levels on estrous cycle and early-pregnancy embryo number in mice (X ± SD, *n* = 5).

DES Dose (mg/kg·BW)		Control	0.02 mg/kg	0.2 mg/kg	2 mg/kg
Body Weight (g)	P1	24.61 ± 1.20 ^b^	24.41 ± 0.72 ^b^	25.51 ± 0.93 ^b^	27.72 ± 1.08 ^a^
	P3	24.58 ± 1.10 ^b^	24.87 ± 0.86 ^b^	25.95 ± 1.34 ^b^	28.14 ± 0.92 ^a^
	P5	29.23 ± 1.47 ^a^	29.04 ± 1.15 ^a^	28.10 ± 0.92 ^a^	28.49 ± 0.56 ^a^
	P7	28.12 ± 0.74 ^a^	29.93 ± 2.66 ^a^	31.19 ± 1.59 ^a^	31.00 ± 2.63 ^a^
	P9	28.42 ± 1.05 ^a^	28.84 ± 0.70 ^a^	29.81 ± 2.80 ^a^	29.62 ± 1.44 ^a^
Estrous Cycle		4.60 ± 0.55 ^a^	5.20 ± 0.76 ^ab^	6.10 ± 0.34 ^b^	7.30 ± 0.48 ^c^
Early-Pregnancy Embryo Number		12.50 ± 1.34 ^a^	9.50 ± 1.16 ^b^	6.00 ± 2.15 ^c^	3.30 ± 0.59 ^d^

Note: Superscript lowercase letters denote statistically significant differences among groups (*p* < 0.05). Groups marked by distinct letters (such as a, b, c, d) differ significantly, while groups sharing letters exhibit no significant difference. Data are displayed as mean values with SD.

## Data Availability

The original contributions presented in this study are included in the article. Further inquiries can be directed to the corresponding author.
